# miR-186-3p attenuates the tumorigenesis of cervical cancer via targeting insulin-like growth factor 1 to suppress PI3K-Akt signaling pathway

**DOI:** 10.1080/21655979.2021.1977053

**Published:** 2021-09-23

**Authors:** Xiurong Lu, Xiao Song, Xiaohui Hao, Xiaoyu Liu, Xianyu Zhang, Na Yuan, Huan Ma, Zhilin Zhang

**Affiliations:** Department of Radiotherapy, The First Affiliated Hospital of Hebei North University, Zhangjiakou, Hebei, China

**Keywords:** Cervical cancer, miR-186-3p, IGF1, PI3K/AKT, tumorigenesis

## Abstract

miR-186-3p acts as a tumor suppressor in various cancers. This study aimed to explore the expression levels of miR-186-3p and its role in cervical cancer. We analyzed the effects of miR-186-3p and insulin-like growth factor 1 (IGF1) on the proliferation, invasion, and apoptosis of cervical cancer cells in vitro by regulating the PI3K/Akt signaling pathway. In cervical cancer tissues and cells, miR-186-3p was downregulated, and IGF1 was upregulated. In addition, miR-186-3p inhibited cell proliferation and invasion and enhanced apoptosis of cervical cancer cells. Moreover, our results showed that miR-186-3p inversely regulated the mRNA expression of IGF1 through direct contact. Knockdown of IGF1 reversed the results of miR-186-3p inhibitor in cervical cancer cells. In addition, the PI3K/Akt signaling pathway was activated by the miR-186-3p inhibitor, although partially arrested by IGF1 knockdown. The PI3K/Akt signaling pathway inhibitor suppressed miR-186-3p inhibitor-stimulated cell proliferation in cervical cancer. In conclusion, miR-186-3p inhibits tumorigenesis of cervical cancer by repressing IGF1, which inactivates the PI3K/Akt signaling pathway, implicating miR-186-3p as a potential new target for the treatment of cervical cancer.

## Introduction

According to cancer statistics reported in 2020, cervical cancer (CC) ranks as the second leading cause of death among women aged 20–39 years suffering from cancer. Approximately 10 patients diagnosed with cancer die from CC per week [1]. The survival rate of most cancer patients has improved, except for patients with the uterine cervix and uterine corpus over the past few decades. Between 1975–1977 and 2006–2012, the survival for CC decreased from 69.1% to 68.8% [2]. Hence, further advances in treatments are crucial to suppress tumor growth, prolong survival, and improve the quality of life of CC patients. Moreover, it has been reported that genetic and epigenetic changes, particularly microRNAs, act in the screening and progression of CC [3].

miRNAs have a significant impact on translation inhibition, mRNA decay, and mRNA deadenylation through the combination with the 3´-untranslated region (3´ UTR) of most protein-coding genes [4,5]. Several studies have shown that miRNAs are involved in the occurrence and development of tumors by regulating the proliferation, differentiation, apoptosis, angiogenesis, metastasis, and metabolism of tumor cells [6]. For instance, overexpression of miR-25-3p significantly promoted the growth and invasion of gastric cancer cells in vitro and regulated the growth of gastric cancer cells in vivo [7]. MiR-214-3p upregulates migration, invasion, and epithelial-mesenchymal transformation of colon cancer cells [8]. MiR-186 has been extensively studied in recent years. However, most studies have focused on miR-186-5p. It has been suggested that miR-186-5p exists as a tumor suppressor factor in CC. Moreover, miR-186-5p downregulation leads to a decline in cell proliferation, migration, and invasion and an increase in apoptosis [9,10]. Regarding miR-186-3p, only one study has identified that it suppresses tumor growth and regulates glycolysis by directly targeting epiregulin in estrogen receptor-positive breast cancer cells [11]. However, the detailed characterization of miR-186-3p in CC is unclear.

The insulin-like growth factor 1 (IGF1) gene is located on chromosome 12q23.2 and consists of seven exons. It encodes proteins that function and have structure similar to insulin, and growth hormones require IGF1 to function [12]. Interest in IGF1 and its effect on carcinogenesis has increased recently. Several studies showed that circulating levels and tissue expression of IGF1 are upregulated in patients with CC [13,14]. IGF1 has been implicated in promoting mitogenic and metastatic cancer cells, which enhances CC invasiveness and proliferation [15,16]. Only one study, which was conducted to analyze the effect of miR-186-5p on IGF1, showed that miR-186-5p induced significant apoptosis of neurons by directly targeting and suppressing IGF1 expression [17]. However, the effect of the interaction between miR-186-3p and IGF1 on tumor growth in CC has not yet been established.

Therefore, this study aimed to explore how miR-186-3p and IGF1 affect CC progression and their possible mechanisms of action. We hypothesized that miR-186-3p might suppress CC by targeting IGF1. In CC, miR-186-3p expression was decreased and IGF1 expression was increased. Further functional experiments confirmed that the inhibitory effect of miR-186-3p on the viability, proliferation, invasion of CC, and the promotion of apoptosis were achieved by targeting and inhibiting IGF1. Our study reveals for the first time the effect of miR-186-3p/IGF1 on CC and its mechanism of action, providing a valuable direction for the diagnosis and treatment of CC.

## Materials and methods

### Tissue samples

Fifty patients with CC aged 28–81 years (mean age, 52 years) were included in this study. The samples were stored at −80°C after liquid nitrogen refrigeration. All study participants provided their samples with informed consent. All experiments followed the ethics committee. The clinicopathological features of all patients are summarized in Table 1.

**Table 1.** The relationship between miR-186-3p or IGF1 expression and clinicopathological characteristics in 50 patients with cervical cancer

Characteristic	N	miR-186-3p expression		P-value	IGF1 expression		P-value
		High (n = 25)	Low (n = 25)		High (n = 25)	Low (n = 25)	
Age (years)				0.382			0.145
≥50	31	17	14		18	13	
<50	19	8	11		7	12	
Tumor size (cm)				0.765			0.371
≥ 5	17	8	9		10	7	
< 5	33	17	16		15	18	
TNM stage				0.004			0.023
I–II	28	19	9		10	18	
III–IV	22	6	16		15	7	
Distant metastasis				0.012			0.031
No	35	22	13		14	21	
Yes	15	3	12		11	4	
Lymph node metastasis				0.011			0.002
No	23	16	7		6	17	
Yes	27	9	18		19	8	
Histological grade				0.003			0.018
Well	18	14	4		5	13	
Moderately/Poorly	32	11	21		20	12	

The P-value was calculated by Chi-square test or Fisher’s exact test.

### Cell culture and treatment

The cell culture conditions were 37°C with 5% CO_2_. The human cervical surface epithelial cell line (HcerEpic), human CC cell lines (HeLa, CaSki, SiHa, and C33A), and embryonic kidney cell line (HEK293T) acquired from ATCC (USA) were cultured in a plate with RPMI-1640 medium (Gibco, USA) containing 10% fetal bovine serum and 1% penicillin/streptomycin (Gibco, USA).

The miR-186-3p inhibitor, miR-186-3p mimic, and paired miRNA negative control (inhibitor-NC and mimic-NC) were designed and synthesized by Shanghai Genechem (Shanghai, China). Before transfection, HeLa and SiHa cells were seeded at a density of 5 × 10^4^ cells/well for 12 h. At approximately 40–60% confluence, the cells were transfected with miR-186-3p mimic, miR-186-3p inhibitor, mimic-NC, or inhibitor-NC using Lipofectamine 3000 (Invitrogen, USA). To knock down the expression of IGF1, specific IGF1 interfering oligonucleotides (si-IGF1) were purchased from Shanghai Jikai Gene Chemistry Co. (Shanghai, China). Then, si-IGF1 was used to treat CC cell lines. Nonspecific control siRNA and reagent controls were used in all the experiments. After transfecting the cells for 48 h, the cell suspension was collected for subsequent experiments. For pathway exploration, LY294002 (Medchem Express, USA) was used as a PI3K inhibitor at a concentration of 50 μM. The cells were pretreated with LY294002 for 2 h.

### Quantitative real-time polymerase chain reaction (qRT-PCR)

miR-186-3p expression levels were assessed by qRT-PCR. Total miRNAs were acquired using a miRNeasy Mini kit (Qiagen, Germany) from CC tissues or cell lines. cDNA was synthesized using the TaqMan MicroRNA Reverse Transcription kit (Applied Biosystems, USA) according to the manufacturer’s instructions. A miRNA-specific TaqMan MiRNA Assay kit (Applied Biosystems, USA) was used for qRT-PCR. The 2-^ΔΔ^Ct method was applied, which was standardized using U6 small nuclear RNA.

For the mRNAs, the expression level of IGF1 was determined by qRT-PCR in the CC tissues and cells. Briefly, total RNA was extracted from tissue samples and cells using TRIzol reagent (Takara, Japan). The RNA content was quantified, and cDNA was synthesized using the Reverse Transcriptase kit (Takara, Japan) following the manufacturer’s instructions. The SYBR Green kit (Takara, China) was used for the determination of cDNA templates. All primers used in this study are listed in Table 2. The 2-^ΔΔCt^ method [18] was used to analyze the mRNA levels of IGF1 with glyceraldehyde-3-phosphate dehydrogenase (GAPDH) as a normalization control.

**Table 2.** PCR primers used in this study

Gene	Primer sequence
IGF1	Forward:5′-TCGCATCTCTTCTATCTGGCCCTGT-′3
	Reverse:5′-GCAGTACATCTCCAGCCTCCTCAGA -′3
GAPDH	Forward:5′-GGAAGGTGAAGGTCGGAG TCA-′3
	Reverse:5′-GTCATTGATGGCAACAATATCCAC T-′3
U6	Forward:5′-CTCGCTTCGGCAGCACA-′3
	Reverse:5′-AACGCTTCACGAATTTGCGT-′3

### Cell proliferation assay

CC cell proliferation was detected using 3 3-(4,5-dimethylthiazol-2-yl)-2,5-diphenyltetrazolium bromide (MTT) [8] and enzyme-linked immunosorbent assay (ELISA) – bromodeoxyuridine (BrdU) assays [19]. Briefly, cells seeded in a specific plate at a density of 4 × 10^4^ cells/well were analyzed at 24, 48, and 72 h after transfection. Every time point of detection followed a 5-h incubation with 20 µL of MTT (KeyGEN BioTECH, China). The absorption degree at 570 nm was examined using an EXL-800 microplate reader (BIOTEK, USA). BrdU incorporation assay was used to analyze cell proliferation using the BrdU Cell Proliferation Assay kit (Cell Signaling Technology, USA). For the assay, cells were incubated with 1X BrdU solution for 6 h at 37°C to induce proliferation and incorporation of BrdU. Thereafter, the labeled cells were cleaned and cultured with detection antibody solution for 1 h. Next, the secondary antibody solution was added, and the plate was incubated for 30 min. The absorbance at 450 nm was read using an EXL-800 microplate reader (BIOTEK, USA).

### Transwell assay

CC cell invasion was measured as described previously [20]. Briefly, serum-free RPMI-1640 medium (Gibco, USA) containing 2 × 10^5^ transfected HeLa and SiHa cells were placed on the upper chamber of the cell culture insert with Matrigel. Thereafter, 600 μL of the complete medium was loaded below the cell-permeable membrane. The cells were incubated for 24 h in 5% CO_2_ at 37°C. The cells gently migrating from the permeable membrane were treated for 30 min in 70% ethanol and 10 min in 0.1% crystal violet. Invasive cells in five random fields were counted using an inverted light microscope.

### Caspase-3 activity assay

Caspase-3 Activity ELISA kit (Cell Signaling Technology, USA) was used to detect caspase-3 activity [21]. Caspase-3 activity was assessed using an ultraviolet spectrophotometer (Thermo, USA) at 405 nm. To conduct this experiment, 6-well plates (1 × 10^5^ cells/well) were used.

### Dual-luciferase reporter assay

The luciferase assay was performed as previously described [22]. HeLa and SiHa cells in the exponential growth period were used for the luciferase assay. The amplified wild-type (Wt) or mutant-type (Mut) IGF1 3´ UTR segment was inserted into the pGL3 luciferase reporter. These reporter plasmids were co-transfected with miR-186-3p into HeLa and SiHa cells using Lipofectamine 3000 (Invitrogen, USA). The Dual-Luciferase Reporter Assay System (Promega, USA) was used for analysis after 72-h-cell incubation.

### RNA-pull down assay

This assay was performed following the method of Lal et al. [23]. Briefly, cells transfected with either biotinylated-miR-186-3p (Bio-miR-186-3p) or negative control oligo (Bio-NC) (25 nM; Roche, USA) were seeded in six-well plates. Thereafter, lysis buffer was utilized for cell lysis, and the cytoplasmic lysate was split into two parts: 95% of the lysate was processed for biotin pull-down assay using streptavidin-coated magnetic beads (Invitrogen, USA), RNA was extracted using the RNeasy Kit (Invitrogen, USA), and the remaining 5% of the lysate was used to extract RNA as an input standard. The levels of mRNA in the miR-186-3p or miR-NC pull-down were analyzed by qRT-PCR. GAPDH mRNA was used to standardize mRNA levels of the target genes. Then, the mRNA gathered by bio-miR-186-3p or bio-NC pulldown was detected by the ratio of the control-normalized pull-down RNA to the control-normalized input levels.

### Western blotting

Western blotting was performed as described previously [24]. Total proteins from each sample were dissolved in radioimmunoprecipitation assay lysis buffer and separated by 12% sodium dodecyl sulfate-polyacrylamide gel electrophoresis. Subsequently, these separated samples were transferred to polyvinylidene difluoride membranes (Millipore, USA). Nonspecific binding was blocked with 5% fat-free milk at room temperature. Primary antibodies, including IGF1 (1:400; Abcam, USA), PI3K (1:1000; Cell Signaling Technology, USA), Akt (1:1000; Cell Signaling Technology, USA), p-Akt (1:1000; Cell Signaling Technology, USA), t-Akt (1:1000; Cell Signaling Technology, USA), and GAPDH (1:1,000; Abcam, USA) were used to detect the corresponding antigen. Following incubation with secondary antibody (1:5000; Cell Signaling Technology, USA) at room temperature for 1 h, protein levels were analyzed.

### Statistical analysis

GraphPad Prism 6 (GraphPad Software, USA) was used for data analysis. All results were expressed as means ± standard deviations (SD). Student’s t-test was used to analyze the differences between two groups. Multiple comparisons were assessed by one-way ANOVA with LSD post hoc test. P < 0.05 was considered statistically significant.

## Results

### Downregulation of miR-186-3p affects CC cell proliferation, invasion and apoptosis

**Figure 1.**
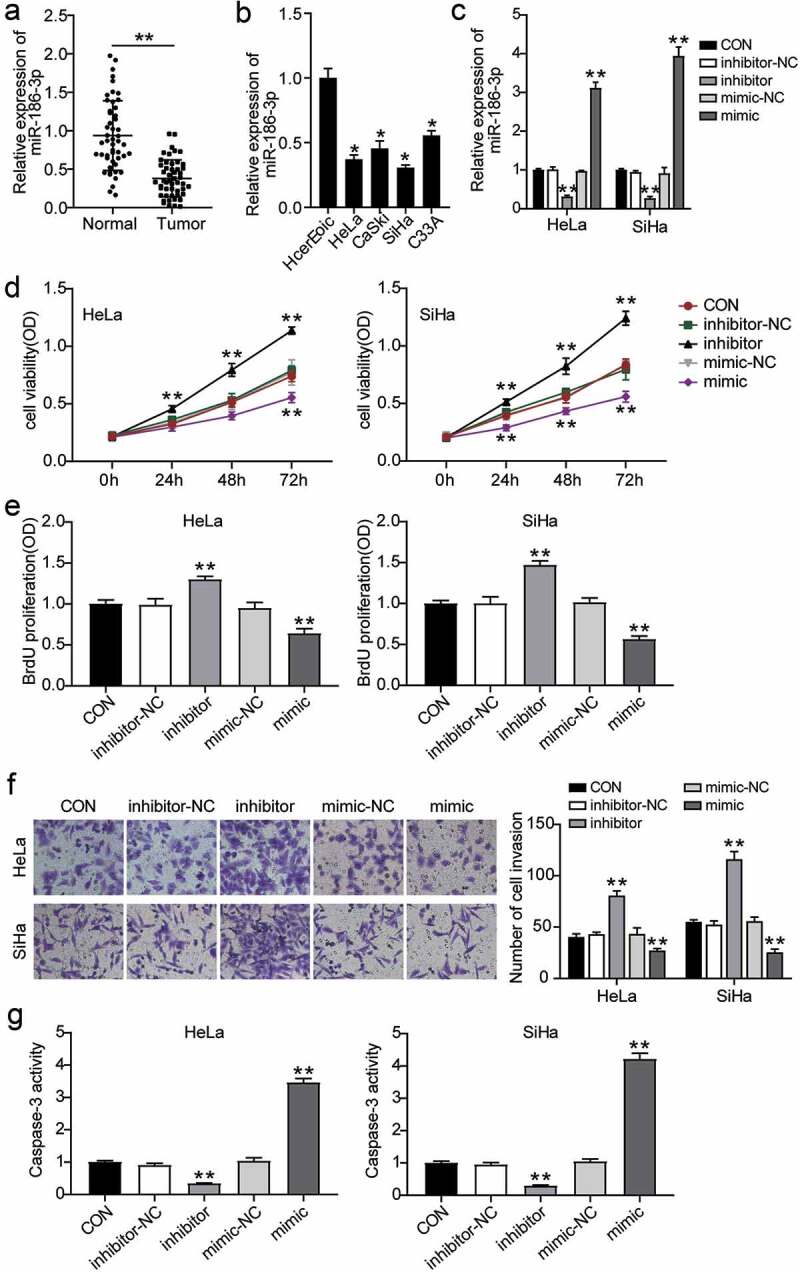
**MiR-186-3p is downregulated and correlated with cell proliferation, invasion and apoptosis of cervical cancer**. (a) MiR-186-3p expression by qRT-PCR in cervical cancer tissues and the adjacent normal tissues. **P < 0.001 vs. adjacent normal tissues. (b) MiR-186-3p expression by qRT-PCR in the human cervical surface epithelial cell line (HcerEpic) and four cervical cancer cell lines (HeLa, CaSki, SiHa, and C33A). **P < 0.001 vs. HcerEpic cell. (c) MiR-186-3p expression by qRT-PCR in the HeLa and SiHa cells transfected with miR-186-3p inhibitor or mimic. **P < 0.001 vs. vs. CON. (d, e) MTT (d) and BrdU incorporation (e) assays of cell viability after HeLa and SiHa cells transfected with miR-186-3p inhibitor or mimic. *P < 0.05; **P < 0.001 vs. CON. (f) Transwell assay in HeLa and SiHa cells transfected with miR-186-3p inhibitor or mimic. Left panel: representative images of the lower chamber (invading cells). Right panel: graph represents number of cell invasion. *P < 0.05; **P < 0.001 vs. CON. (g) Caspase-3 activity ELISA assay in HeLa and SiHa cells transfected with miR-186-3p inhibitor or miR-NC. **P < 0.001 vs. CON. MiR, microRNA; CON, control; NC, negative control

To explore the effect of miR-186-3p on CC, we detected the expression of miR-186-3p in CC tissues and cell lines and analyzed the effect of miR-186-3p on the CC cell viability, proliferation, migration, and apoptosis in vitro through MTT assay, BrdU assay, transwell assay, and caspase-3 activity, respectively. The antitumor effects of miR-186-3p in CC have been clarified. Initially, miR‑186-3p levels in CC tissues and CC cells were quantified for the preliminary investigation of the role of miR-186-3p in CC pathological processes. As shown in Figure 1a, miR-186-3p levels reduced (over 50%) in the CC tissues compared to the adjacent normal tissues. The median expression level of miR-186-3p was used as a cutoff point to divide all 50 patients into two groups: CC patients who expressed miR-186-3p at levels less than the cutoff value were assigned to the low expression group (n = 25), and those with expression levels above the cutoff value were assigned to the high expression group (n = 25). The relationship between miR-186-3p expression levels and different clinicopathological factors is shown in Table 1. Decreased miR-186-3p expression in CC was significantly associated with the tumor node metastasis (TNM) stage, distant metastasis, lymph node metastasis, and histological grade. However, no significant correlation was observed between miR-186-3p expression and other clinicopathological variables, such as age and tumor size. In addition, we observed that the miR-186-3p level in four CC cell lines was significantly decreased by approximately 50% compared to that in HcerEpic cells (Figure 1b). As a result, miR-186-3p exhibited the lowest level in HeLa and SiHa cell lines, which were selected for the following experiments.

Furthermore, qRT-PCR analysis confirmed the transfection efficiency and showed that miR-186-3p level was repressed by treatment with miR-186-3p inhibitor and enhanced by miR-186-3p mimic (Figure 1c). The MTT and BrdU incorporation assays demonstrated that the miR-186-3p inhibitor enhanced CC cell proliferation, whereas the miR-186-3p mimic suppressed it (Figure 1d and 1e). Moreover, miR-186-3p downregulation increased the invasiveness of CC cells in vitro, and miR-186-3p upregulation showed the opposite effect (figure 1f). Caspase-3 activity was also evaluated. The inhibitor of miR-186-3p decreased caspase-3 activity, and a miR-186-3p mimic increased caspase-3 activity, a critical executioner participated in ‘extrinsic’ and ‘intrinsic’ apoptosis (Figure 1g). These findings demonstrated that upregulation of miR-186-3p inhibits tumorigenesis in CC, and downregulation of miR-186-3p promotes tumor progression.

### MiR-186-3p targets IGF1 in CC cells

**Figure 2.**
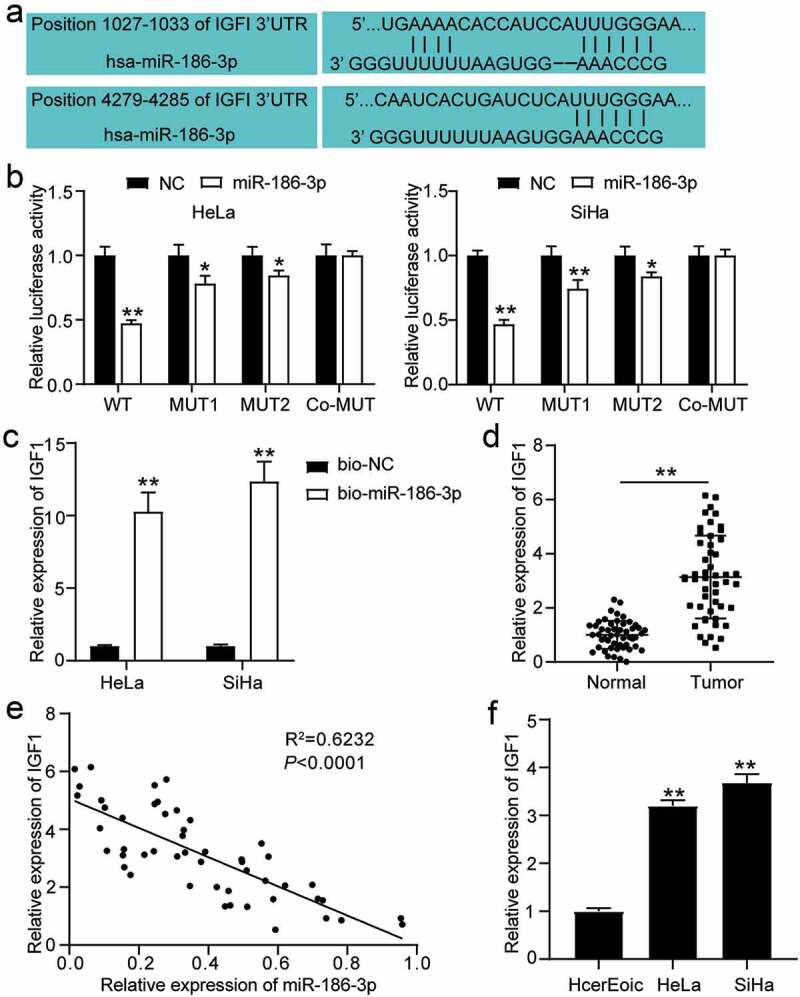
**IGF1 is a direct target of miR-186-3p in cervical cancer cell**. (a) IGF1 was predicted as miR-186-3p target. Top and bottom, predicted two miR-186-3p binding site in the 3′ UTR of IGF1. (b) Dual-luciferase reporter assay. NC RNA was set to 1.0 in each experiment, and the luciferase activity with miR-186-3p was normalized relative to NC RNA. *P < 0.05; **P < 0.001 vs. NC. (c) RNA-pull down assay. qRT-PCR of IGF1 expression in HeLa and SiHa cells transfected with Bio-NC and Bio-miR186-3p. **P < 0.001 vs. bio-NC. (d) Comparison of IGF1 expression in the cervical cancer tissues and the adjacent normal tissues. **P < 0.01 vs. adjacent normal tissues. (e) Pearson’s correlation analyses showing a negative correlation of miR-186-3p and IGF1 mRNA levels in cervical cancer tissues. (f) qRT-PCR of IGF1 in HcerEpic, HeLa and SiHa cells. **P < 0.001 vs. HcerEpic cells. IGF1, insulin-like growth factor 1; 3ʹUTR, 3ʹuntranslated region; miR, microRNA; NC, negative control; WT, wild-type; MUT, mutant-type; bio-NC, biotinylated- negative control oligo

To elucidate the mechanism of miR-186-3p in CC, luciferase and RNA pull-down assays were used to analyze the binding relationship between miR-186-3p and IGF1. The results showed that miR-186-3p inhibited IGF1. Bioinformatics analysis was then used to identify the possible molecular targets of miR-186-3p. The prediction results showed that the 3´ UTR of IGF1 contained two binding sites for miR-186-3p (Figure 2a). To determine whether IGF1 was the direct target of miR-186-3p, we constructed luciferase reporters containing the IGF1-3´ UTR with a conserved miR-186-3p binding sequence or two mutated binding sequences of miR-186-3p. As a result, miR-186-3p significantly attenuated the luciferase activity of the wild-type but not the 3´ UTR of IGF1 two mutated binding sequences of miR-186-3p (Figure 2b). To further confirm the physical binding of IGF1 with miR-186-3p, we conducted an RNA pull-down assay in HeLa and SiHa cells transfected with Bio-miR186-3p. As displayed in Figure 2c, IGF1 mRNA levels were significantly enriched by bio-miR186-3p pulldown compared to bio-NC. These results suggest that miR-186-3p physically interacts with IGF1.

Thereafter, we examined the expression of IGF1 in CC tissues and cell lines. It was demonstrated that the IGF1 level was enriched in the CC tissues compared to the adjacent normal tissues (Figure 2d). Furthermore, IGF1 upregulation in CC was significantly associated with the TNM stage, distant metastasis, lymph node metastasis, and histological grade, except with age or tumor size (Table 1). In addition, IGF1 expression was significantly higher in HeLa and SiHa cells (figure 2e). IGF1 levels were negatively correlated with miR-186-3p expression in CC tissues (Figure 2f). In summary, miR-186-3p negatively regulated IGF1 expression through binding to the 3´ UTR sequence in CC.

### IGF1 mediates the functional effects of miR-186-3p in CC cells

**Figure 3.**
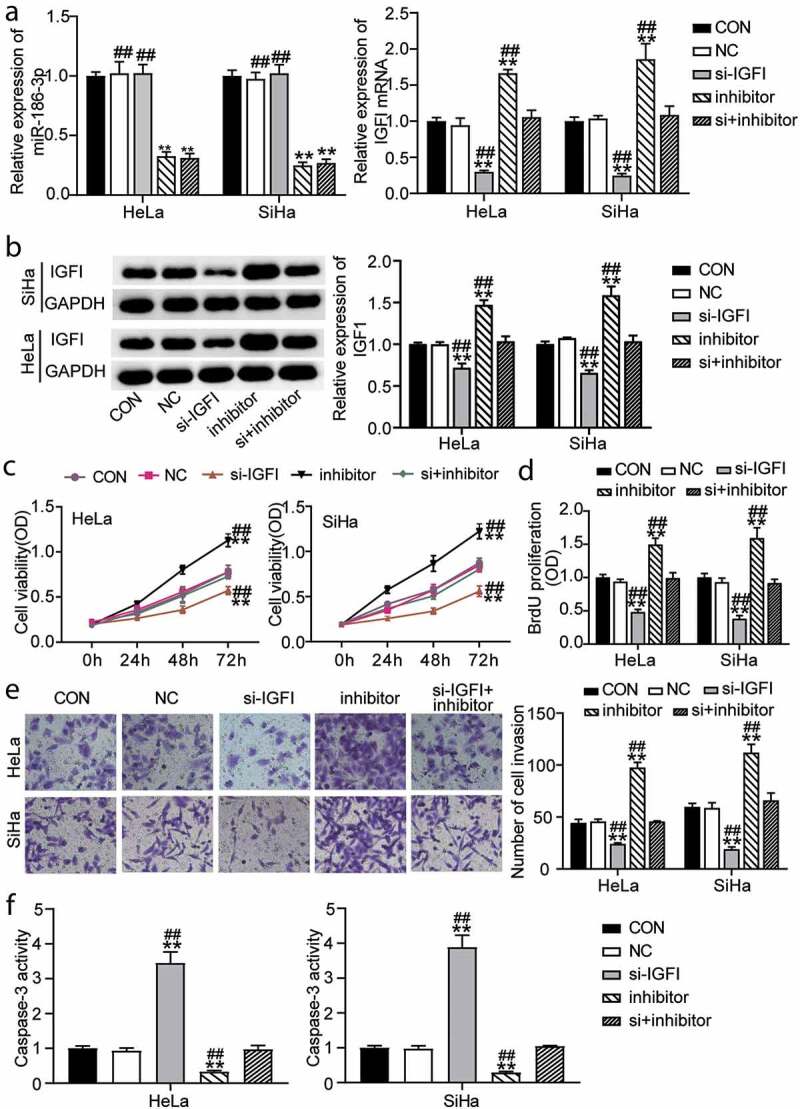
**IGF1 mediates the functional effects of miR-186-3p on cervical cancer cell**. (a) qRT-PCR of MiR-186-3p (left panel) and IGF1 (right panel) expression in HeLa and SiHa cells transfected with si-IGF1 or miR-186-3p inhibitor. (b) western blot analyses of IGF1 expression in miR-186-3p inhibitor- or si-IGF1-transfected HeLa and SiHa cells. (c, d) MTT (c) and BrdU incorporation (d) assays of cell viability after HeLa and SiHa cells transfected with si-IGF1 or miR-186-3p inhibitor. (e) Transwell assay in HeLa and SiHa cells transfected with si-IGF1 or miR-186-3p inhibitor. Left panel: representative images of the lower chamber (invading cells). Right panel: graph represents number of cell invasion. (f) Caspase-3 activity ELISA assay in HeLa and SiHa cells transfected with si-IGF1 or miR-186-3p inhibitor. *P < 0.05; **P < 0.001 vs. CON. ^#^*P* < 0.05; ^##^*P* < 0.001 vs. si+inhibitor. CON, control; NC, negative control; si-, small interfering RNA; IGF1, insulin-like growth factor 1; miR-, microRNA

To further study the effect of miR-186-3p on the regulation of IGF1 in CC cells, we analyzed the changes in viability, proliferation, invasion, and apoptosis of CC cells after co-low expression of miR-186-3p and IGF1. The results showed that the effect of miR-186-3p on CC cells was mediated by GINS2. First, to determine whether IGF1 contributes to the miR-186-3p reaction against CC, si-IGF1 and miR-186-3p inhibitors were co-transfected into HeLa and SiHa cells. The mRNA levels of IGF-1 and miR-186-3p were measured using qRT-PCR. Western blotting was performed to detect the protein levels of IGF-1. After transfection, the relative expression of IGF1 and miR-186-3p in si-IGF1-transfected and miR-186-3p inhibitor-transfected HeLa and SiHa cells significantly decreased, indicating that miR-186-3p and IGF1 expression was effectively suppressed (Figure 3a and 3 b). The proliferation of CC cell lines was significantly inhibited in the si-IGF1 group. However, cell proliferation was significantly upregulated by co-transfection of miR-186-3p inhibitor with si-IGF1 compared to CC cells transfected with si-IGF1 (Figure 3c and 3d). Transwell assays revealed that the invasion capacity of the CC cells was significantly suppressed by transfection with si-IGF1. Inhibition of miR-186-3p promoted CC cell invasion via IGF1 inhibition (Figure 3e). Furthermore, figure 3f shows that miR-186-3p inhibition suppressed caspase-3 activity, whereas additional IGF1 downregulation partially promoted caspase-3 activity in HeLa and SiHa cells. Therefore, these data indicate that miR-186-3p inhibits CC cell growth and invasion and promotes apoptosis by suppressing IGF1 expression.

### MiR-186-3p suppress PI3K/Akt pathway by downregulating IGF1 expression

**Figure 4.**
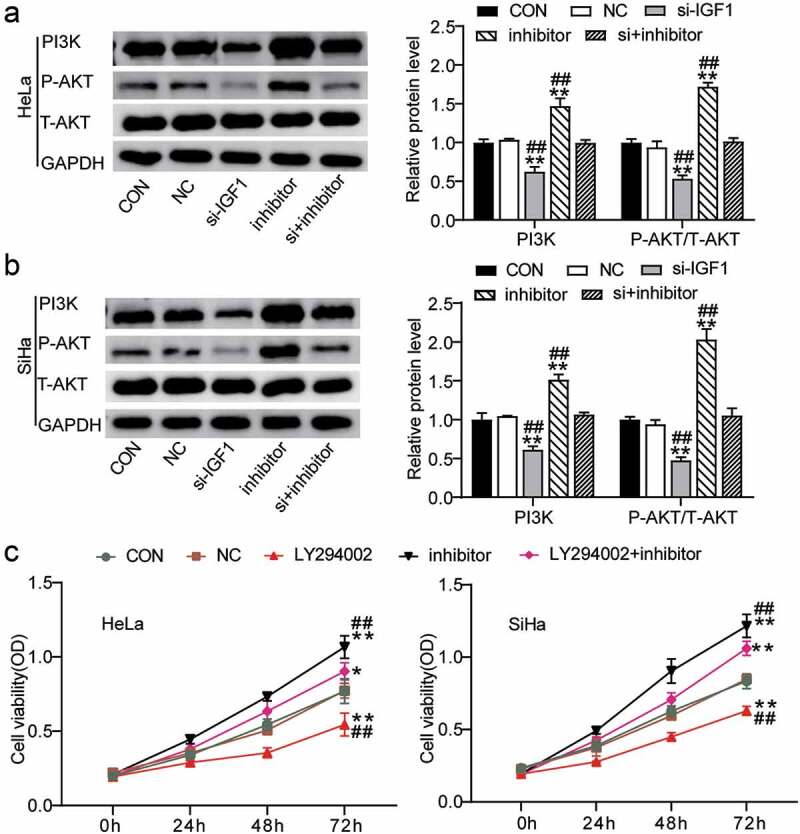
**MiR-186-3p suppress PI3K/Akt pathway through downregulating IGF1 expression**. (a, b) western blot analyses of PI3K, P-AKT and T-AKT expression in miR-186-3p inhibitor- or si-IGF1-transfected HeLa (a) and SiHa (b) cells. *P < 0.05; **P < 0.001 vs. CON. ^#^*P* < 0.05; ^##^*P* < 0.001 vs. si+inhibitor. (c) MTT assays of cell viability after HeLa and SiHa cells pretreated by PI3K signaling inhibitor (LY294002) following transfection with miR-186-3p inhibitor. *P < 0.05; **P < 0.001 vs. CON. ^#^*P* < 0.05; ^##^*P* < 0.001 vs. LY294002+ inhibitor. P-AKT, phosphorylated-AKT. T-AKT, total-AKT; CON, control; NC, negative control; si-, small interfering RNA; IGF1, insulin-like growth factor 1

**Figure 5.**
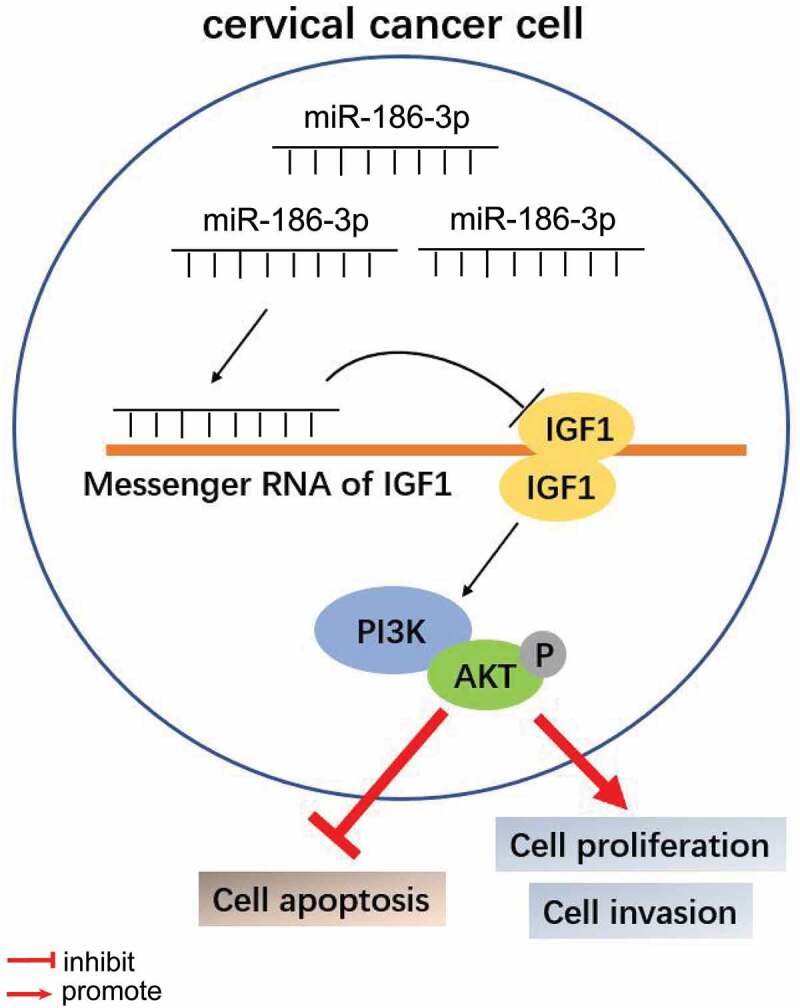
Model explaining the antitumor effect of miR-186-3p in cervical cancer

Next, the downstream signaling pathways of miR-186-3p/IGF1 affecting the biological functions of CC cells were identified. Western blot and MTT analysis showed that silencing miR-186-3p or IGF1 inhibited PI3K/Akt pathway activation. Previous studies have shown that the inactivation of the PI3K/Akt pathway inhibits carcinogenesis and the development of cancer [25,26]. In addition, the PI3K/AKT pathway is an indispensable pathway mediated by numerous cellular signals, including IGF-1 [27]. Hence, we further investigated whether miR-186-3p/IGF1 exerts its function via the PI3K/Akt signaling pathway. As shown in Figure 4a and 4b, the protein expression of PI3K and phosphorylation of AKT were decreased by IGF1 knockdown. In contrast, the miR-186-3p inhibitor upregulated the protein levels of PI3K and phosphorylation of AKT. In addition, si-IGF1 suppressed the activation of the PI3K/AKT pathway induced by the miR-186-3p inhibitor. Therefore, we examined whether the PI3K signaling inhibitor LY294002 could suppress CC cell proliferation. As shown in Figure 4c, the results of the MTT assays indicated that CC cell proliferation was increased after miR-186-3p downregulation. Moreover, LY294002 suppressed the increase in CC cell proliferation following miR-186-3p inhibition. These results revealed that miR-186-3p prevents cell proliferation through the inactivation of the PI3K/AKT signaling pathway via inhibition of IGF1 in CC cell lines (Figure 5).

## Discussion

miRNAs are regarded as critical regulators of cancer. Several studies have reported that miR-186 is downregulated in numerous types of human cancers, including non-small cell lung cancer [28], colon carcinoma [29], acute myeloid leukemia [30], esophageal squamous cell carcinoma [31], bladder cancer [32], gastric cancer [33], and hepatocellular carcinoma [34]. In addition, cell life processes in cancers are affected by miR-186 [35]. In non-small cell lung cancer, miR-186 was upregulated and targeted Rho-associated protein kinase 1 to suppress the proliferation and migration of cancer cells [36]. In bladder cancer, upregulated miR-186 decreased the proliferation and invasion of cells through the downregulation of nucleosome‑binding protein 1 [32]. In CC, high expression of miR-186 targets Kazrin-F to promote apoptosis and suppress cell proliferation, colony formation, migration, and invasion of CC cells [32]. Zhang et al. [37] found that miR-186 reversed the function of the INK4 locus (ANRIL), leading to the suppression of CC. The above data support miR-186 as a key regulator and candidate therapeutic target in the tumor treatment. As for miR-186-3p, only one study has identified that miR-186-3p suppresses tumor growth and regulates glycolysis by directly targeting epiregulin in estrogen receptor-positive breast cancer [11]. Based on the findings stated above, we speculated that miR-186-3p plays pivotal roles in modulating CC pathological processes. Our data demonstrated the downregulation of miR-186-3p in CC and CC cell lines. In functional studies, miR-186-3p downregulation remarkably contributed to CC cell proliferation. However, miR-186-3p suppression significantly inhibited apoptosis and reduced the expression of apoptosis-related factors. These results suggest that miR-186-3p can be regarded as an antitumor agent in CC.

To further determine the mechanisms and targets of miR-186-3p, we performed bioinformatics, dual-luciferase reporter, and biotinylated-miRNA pulldown assays to confirm the predicted binding ability of miR-186-3p on IGF-1. Previous studies have shown that IGF1, a mitogenic and anti-apoptotic peptide, is upregulated in CC [13]. IGF1 stimulates cell growth and invasiveness in CC in a dose-dependent manner [15]. Furthermore, IGF1 inhibition induced an increase in the G2M/S fraction, increased apoptosis, and decreased invasive ability of CC stem cells [38], suggesting that IGF1 has a significant effect on the progression of CC. Moreover, additional studies have shown that IGF1 expression is upregulated in other cancers (including hepatocellular carcinoma), predicting a lower 10-year survival rate [39]. Similar to the previous study, our observations showed that IGF1 significantly decreased in the CC tissues and cells. In addition, IGF1 was negatively correlated with miR-186-3p expression in cancer tissues. Inhibition of miR-186-3p significantly increased IGF1 expression in CC. Furthermore, miR-186-3p reduction promoted CC cell proliferation and invasiveness and inhibited apoptosis. IGF1 knockdown displayed the opposite effects. Our observation revealed for the first time that miR-186-3p acts as a carcinoma inhibitor in CC, at least in part, through the negative regulation of IGF1.

To further elucidate the possible molecular mechanisms, we explored the PI3K/Akt signaling pathway, which is activated by IGF1 and regulates fundamental cellular processes, including cell proliferation, differentiation, apoptosis, metastasis, and epithelial-mesenchymal transition [40]. A recent report found that the PI3K/Akt pathway was activated in endometriosis, and the expression of p-Akt and p-PI3K was upregulated [41]. In addition, other studies have shown the importance of the PI3K/Akt pathway in the progression of tumors, especially CC [42,43]. Recent studies have indicated that CC cells express high levels of PI3K, Akt, and phosphorylated Akt [44–46]. Moreover, treatment with PI3K/Akt pathway inhibitors prevented cell proliferation [47], induced apoptosis [48], and restored metastasis [49] in CC. These results illustrated that the PI3K/Akt pathway may be a novel biomarker for predicting the prognosis of CC and a potential target for developing new treatment for such tumors [50]. This study found that inhibition of miR-186-3p significantly increased PI3K expression and phosphorylated AKT in CC cell lines. Moreover, this effect was reversed by the addition of IGF1 knockdown. Inhibition of miR-186-3p remarkably enhanced CC cell proliferation. LY294002 suppressed the PI3K/AKT signaling pathway and decreased the tumorigenic impact of the miR-186-3p repressor on CC cell proliferation. These findings strongly suggest that the negative function of miR-186-3p in the tumorigenesis of CC is mediated by the PI3K/Akt pathway. A possible explanation might be related to the fact that miR-186-3p targets and binds to IGF1 and inhibits the activation of specific receptor tyrosine kinases (RTKs), thereby blocking the PI3K/AKT pathway, leading to increased apoptosis and decreased tumor formation, as shown in the present study (Figure 5).

## Conclusions

In conclusion, our study suggests that miR-186-3p inhibits proliferation, promotes apoptosis of CC cells, and inactivates the PI3K/AKT signaling pathway by targeting IGF1. In addition, miR-186-3p serves as a potentially promising target for combating CC in the future. However, further in vivo studies are needed to confirm the functions of miR-186-3p in CC.

## Data Availability

The datasets used and/or analyzed during the current study are available from the corresponding author on reasonable request.
